# 5-Fluoro-1-(penta­noyl)pyrimidine-2,4(1*H*,3*H*)-dione

**DOI:** 10.1107/S1600536808004418

**Published:** 2008-02-22

**Authors:** Hans-Joachim Lehmler, Sean Parkin

**Affiliations:** a Department of Occupational and Environmental Health, University of Iowa, 100 Oakdale Campus, 124 IREH, Iowa City, IA 52242-5000, USA; b Department of Chemistry, University of Kentucky, Lexington, KY 40506-0055, USA

## Abstract

The penta­noyl group and the 5-fluoro­uracil moiety of the title compound, C_9_H_11_FN_2_O_3_, are essentially coplanar, with the penta­noyl carbonyl group oriented towards the ring CH group and away from the nearer ring carbonyl group. In the crystal structure, two inversion-related mol­ecules form a dimer structure, in which two N—H⋯O hydrogen bonds generate an inter­molecular *R*_2_^2^(8) ring. In addition, there are intra- and inter­molecular C—H⋯O inter­actions.

## Related literature

For similar 5-fluoro­pyrimidine-2,4(1*H*,3*H*)-dione structures with N1-acyl substituents, see: Beall *et al.* (1997 ▶); Jiang *et al.* (1988 ▶); Lehmler & Parkin (2000 ▶). For related literature, see: Roberts & Sloan (1999 ▶).
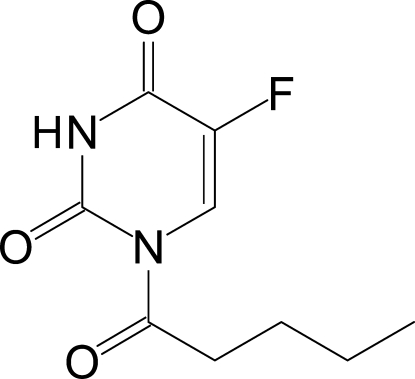


## Experimental

### 

#### Crystal data

C_9_H_11_FN_2_O_3_
*M* *_r_* = 214.20
Triclinic, 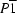

*a* = 5.3165 (2) Å
*b* = 9.3986 (4) Å
*c* = 10.1895 (5) Å
α = 96.000 (3)°
β = 100.957 (3)°
γ = 105.539 (3)°
*V* = 475.04 (4) Å^3^
*Z* = 2
Mo *K*α radiation
μ = 0.13 mm^−1^
*T* = 87.8 (2) K
0.30 × 0.30 × 0.03 mm

#### Data collection

Nonius KappaCCD diffractometer
Absorption correction: multi-scan (*SCALEPACK*; Otwinowski & Minor, 1997 ▶) *T*_min_ = 0.963, *T*_max_ = 0.996
12409 measured reflections
2167 independent reflections
1727 reflections with *I* > 2σ(*I*)
*R*_int_ = 0.037

#### Refinement

*R*[*F* ^2^ > 2σ(*F* ^2^)] = 0.047
*wR*(*F* ^2^) = 0.092
*S* = 1.02
2167 reflections
137 parameters
H-atom parameters constrained
Δρ_max_ = 0.23 e Å^−3^
Δρ_min_ = −0.24 e Å^−3^

### 

Data collection: *COLLECT* (Nonius, 1998 ▶); cell refinement: *SCALEPACK* (Otwinowski & Minor, 1997 ▶); data reduction: *DENZO-SMN* (Otwinowski & Minor, 1997 ▶); program(s) used to solve structure: *SHELXS97* (Sheldrick, 2008 ▶); program(s) used to refine structure: *SHELXL97* (Sheldrick, 2008 ▶); molecular graphics: *XP* in *SHELXTL* (Sheldrick, 2008 ▶); software used to prepare material for publication: *SHELXL97* and local procedures.

## Supplementary Material

Crystal structure: contains datablocks I, global. DOI: 10.1107/S1600536808004418/at2544sup1.cif

Structure factors: contains datablocks I. DOI: 10.1107/S1600536808004418/at2544Isup2.hkl

Additional supplementary materials:  crystallographic information; 3D view; checkCIF report

## Figures and Tables

**Table 1:** Hydrogen-bond geometry (Å, °)

*D*—H⋯*A*	*D*—H	H⋯*A*	*D*⋯*A*	*D*—H⋯*A*
N3—H3⋯O4^i^	0.88	1.99	2.8588 (16)	170
C6—H6⋯O7	0.95	2.28	2.6102 (17)	100
C6—H6⋯O7^ii^	0.95	2.34	3.2266 (19)	154

Symmetry codes: (i) 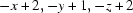; (ii) 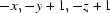.

**Fig. 1.**
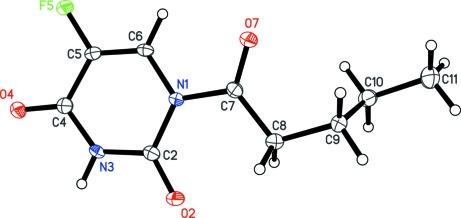
View of the title compound showing the atom-labelling scheme. Displacement ellipsoids are drawn at the 50% probability level.

## supplementary crystallographic information

### Comment

Despite the potential pharmaceutical application of acyl-5-fluorouracil prodrugs, the crystal structures of only three acyl derivatives have been reported (Beall *et al.*, 1997; Jiang *et al.*, 1988; Lehmler & Parkin, 2000). We herein describe the crystal structures of another acyl-5-fluorouracil prodrug, 5-fluoro-1-(1-oxopentyl)-2,4(1*H*,3H)-pyrimidinedione.

The molecular structures of the title compound and the other 1-acyl-5-fluorouracil derivatives are very similar. Specifically, the 1-acyl group and the 5-fluorouracil moiety are almost coplanar, with the C7?O7 carbonyl group oriented towards the C6—H group and away from the C2?O2 group in all four crystal structures. The C6—N1—C7—O7 dihedral angle of all 1-acyl-5-fluorouracil derivatives is comparable and ranges from 1.6 to 17.3° (Beall *et al.*, 1997; Jiang *et al.*, 1988; Lehmler & Parkin, 2000). In the crystal structure, two inversion-related molecules form a dimer structure, in which two N—H···O hydrogen bonds generate an intermolecular *R*_2_^2^(8) ring. In addition, there are C—H···O type-intra and intermolecular interactions.

### Experimental

5-Fluoro-1-(1-oxopentyl)-2,4(1*H*,3H)-pyrimidinedione was synthesized by acylation of 5-fluorouracil with pentanoyl chloride and recrystallized from diethylether at 253 K (Beall *et al.*, 1997; Lehmler & Parkin, 2000; Roberts & Sloan, 1999).

### Refinement

H atoms were found in difference Fourier maps and subsequently placed in idealized positions with constrained C—H distances of 0.98 Å (RCH_3_), 0.99 Å (*R*_2_CH_2_), 0.95 Å (C_Ar_H) and 0.88 Å (NH) with *U*_iso_(H) values set to either 1.2*U*_eq_ or 1.5*U*_eq_ (RCH_3_ only) of the attached atom.

### Crystal data

**Table:**

C_9_H_11_FN_2_O_3_	*Z* = 2
*M**_r_* = 214.20	*F*_000_ = 224
Triclinic, *P*1	*D*_x_ = 1.497 Mg m^−^^3^
Hall symbol: -P 1	Mo *K*α radiation λ = 0.71073 Å
*a* = 5.3165 (2) Å	Cell parameters from 6994 reflections
*b* = 9.3986 (4) Å	θ = 1.0–27.5º
*c* = 10.1895 (5) Å	µ = 0.13 mm^−^^1^
α = 96.000 (3)º	*T* = 87.8 (2) K
β = 100.957 (3)º	Irregular plate, colourless
γ = 105.539 (3)º	0.30 × 0.30 × 0.03 mm
*V* = 475.04 (4) Å^3^	

### Data collection

**Table:**

Nonius KappaCCD diffractometer	2167 independent reflections
Radiation source: fine-focus sealed tube	1727 reflections with *I* > 2σ(*I*)
Monochromator: graphite	*R*_int_ = 0.037
Detector resolution: 18 pixels mm^-1^	θ_max_ = 27.5º
*T* = 87.8(2) K	θ_min_ = 2.1º
ω scans at fixed χ = 55°	*h* = −6→6
Absorption correction: multi-scan(SCALEPACK; Otwinowski & Minor, 1997)	*k* = −12→12
*T*_min_ = 0.963, *T*_max_ = 0.996	*l* = −13→13
12409 measured reflections	

### Refinement

**Table:**

Refinement on *F*^2^	Secondary atom site location: difference Fourier map
Least-squares matrix: full	Hydrogen site location: inferred from neighbouring sites
*R*[*F*^2^ > 2σ(*F*^2^)] = 0.047	H-atom parameters constrained
*wR*(*F*^2^) = 0.092	*w* = 1/[σ^2^(*F*_o_^2^) + (0.0269*P*)^2^ + 0.2309*P*] where *P* = (*F*_o_^2^ + 2*F*_c_^2^)/3
*S* = 1.02	(Δ/σ)_max_ < 0.001
2167 reflections	Δρ_max_ = 0.23 e Å^−^^3^
137 parameters	Δρ_min_ = −0.24 e Å^−^^3^
Primary atom site location: structure-invariant direct methods	Extinction correction: none

### Special details

**Table:**

Geometry. All e.s.d.'s (except the e.s.d. in the dihedral angle between two l.s. planes) are estimated using the full covariance matrix. The cell e.s.d.'s are taken into account individually in the estimation of e.s.d.'s in distances, angles and torsion angles; correlations between e.s.d.'s in cell parameters are only used when they are defined by crystal symmetry. An approximate (isotropic) treatment of cell e.s.d.'s is used for estimating e.s.d.'s involving l.s. planes.
Refinement. Refinement of *F*^2^ against ALL reflections. The weighted *R*-factor *wR* and goodness of fit *S* are based on *F*^2^, conventional *R*-factors *R* are based on *F*, with *F* set to zero for negative *F*^2^. The threshold expression of *F*^2^ > 2σ(*F*^2^) is used only for calculating *R*-factors(gt) *etc*. and is not relevant to the choice of reflections for refinement. *R*-factors based on *F*^2^ are statistically about twice as large as those based on *F*, and *R*- factors based on ALL data will be even larger.

### Fractional atomic coordinates and isotropic or equivalent isotropic displacement parameters (Å^2^)

**Table:**

	*x*	*y*	*z*	*U*_iso_*/*U*_eq_	
N1	0.4196 (2)	0.37039 (13)	0.62262 (12)	0.0131 (3)	
O2	0.7435 (2)	0.24634 (12)	0.65488 (10)	0.0208 (3)	
C2	0.6455 (3)	0.33911 (17)	0.69638 (15)	0.0146 (3)	
N3	0.7532 (2)	0.42493 (13)	0.82378 (12)	0.0149 (3)	
H3	0.8913	0.4049	0.8723	0.018*	
O4	0.7890 (2)	0.60838 (12)	0.99779 (10)	0.0176 (3)	
C4	0.6725 (3)	0.53737 (16)	0.88432 (15)	0.0148 (3)	
F5	0.34305 (17)	0.66618 (10)	0.85412 (8)	0.0198 (2)	
C5	0.4391 (3)	0.56025 (16)	0.80080 (15)	0.0142 (3)	
C6	0.3224 (3)	0.48168 (16)	0.67833 (14)	0.0135 (3)	
H6	0.1698	0.5013	0.6273	0.016*	
O7	0.1183 (2)	0.35259 (12)	0.42664 (10)	0.0189 (3)	
C7	0.2850 (3)	0.30070 (16)	0.48388 (15)	0.0139 (3)	
C8	0.3615 (3)	0.17280 (17)	0.41877 (15)	0.0162 (3)	
H8A	0.3494	0.0949	0.4775	0.019*	
H8B	0.5500	0.2088	0.4108	0.019*	
C9	0.1823 (3)	0.10375 (17)	0.27854 (15)	0.0170 (3)	
H9A	0.1644	0.1853	0.2267	0.020*	
H9B	0.2710	0.0418	0.2302	0.020*	
C10	−0.0962 (3)	0.00735 (17)	0.28048 (16)	0.0192 (4)	
H10A	−0.1828	0.0668	0.3330	0.023*	
H10B	−0.0809	−0.0788	0.3262	0.023*	
C11	−0.2706 (3)	−0.0500 (2)	0.13778 (17)	0.0266 (4)	
H11A	−0.2890	0.0351	0.0929	0.040*	
H11B	−0.4482	−0.1117	0.1427	0.040*	
H11C	−0.1868	−0.1103	0.0860	0.040*	

### Atomic displacement parameters (Å^2^)

**Table:**

	*U*^11^	*U*^22^	*U*^33^	*U*^12^	*U*^13^	*U*^23^
N1	0.0116 (6)	0.0140 (6)	0.0139 (6)	0.0054 (5)	0.0013 (5)	0.0013 (5)
O2	0.0197 (6)	0.0233 (6)	0.0206 (6)	0.0133 (5)	−0.0006 (5)	−0.0007 (5)
C2	0.0119 (7)	0.0156 (8)	0.0167 (8)	0.0044 (6)	0.0029 (6)	0.0047 (6)
N3	0.0117 (6)	0.0165 (7)	0.0155 (7)	0.0060 (5)	−0.0016 (5)	0.0023 (5)
O4	0.0164 (6)	0.0196 (6)	0.0153 (6)	0.0061 (5)	0.0002 (4)	0.0007 (5)
C4	0.0141 (8)	0.0139 (8)	0.0170 (8)	0.0031 (6)	0.0050 (6)	0.0047 (6)
F5	0.0199 (5)	0.0208 (5)	0.0193 (5)	0.0116 (4)	0.0009 (4)	−0.0027 (4)
C5	0.0142 (7)	0.0139 (8)	0.0170 (8)	0.0069 (6)	0.0049 (6)	0.0033 (6)
C6	0.0106 (7)	0.0152 (8)	0.0166 (8)	0.0062 (6)	0.0037 (6)	0.0043 (6)
O7	0.0197 (6)	0.0210 (6)	0.0163 (6)	0.0108 (5)	−0.0009 (5)	0.0011 (5)
C7	0.0115 (7)	0.0153 (8)	0.0149 (7)	0.0030 (6)	0.0035 (6)	0.0036 (6)
C8	0.0159 (8)	0.0166 (8)	0.0172 (8)	0.0066 (6)	0.0035 (6)	0.0037 (6)
C9	0.0175 (8)	0.0176 (8)	0.0156 (8)	0.0064 (7)	0.0026 (6)	−0.0006 (6)
C10	0.0189 (8)	0.0175 (8)	0.0219 (8)	0.0066 (7)	0.0051 (7)	0.0029 (7)
C11	0.0242 (9)	0.0236 (9)	0.0270 (9)	0.0045 (7)	0.0013 (7)	−0.0024 (7)

### Geometric parameters (Å, °)

**Table:**

N1—C6	1.3999 (18)	C7—C8	1.499 (2)
N1—C2	1.4093 (19)	C8—C9	1.526 (2)
N1—C7	1.4526 (18)	C8—H8A	0.9900
O2—C2	1.2084 (17)	C8—H8B	0.9900
C2—N3	1.3837 (18)	C9—C10	1.520 (2)
N3—C4	1.3743 (19)	C9—H9A	0.9900
N3—H3	0.8800	C9—H9B	0.9900
O4—C4	1.2291 (17)	C10—C11	1.523 (2)
C4—C5	1.446 (2)	C10—H10A	0.9900
F5—C5	1.3462 (16)	C10—H10B	0.9900
C5—C6	1.325 (2)	C11—H11A	0.9800
C6—H6	0.9500	C11—H11B	0.9800
O7—C7	1.2077 (17)	C11—H11C	0.9800
			
C6—N1—C2	120.42 (12)	C9—C8—H8A	109.1
C6—N1—C7	115.53 (12)	C7—C8—H8B	109.1
C2—N1—C7	123.89 (12)	C9—C8—H8B	109.1
O2—C2—N3	121.00 (13)	H8A—C8—H8B	107.9
O2—C2—N1	124.45 (13)	C10—C9—C8	114.16 (12)
N3—C2—N1	114.55 (13)	C10—C9—H9A	108.7
C4—N3—C2	128.41 (13)	C8—C9—H9A	108.7
C4—N3—H3	115.8	C10—C9—H9B	108.7
C2—N3—H3	115.8	C8—C9—H9B	108.7
O4—C4—N3	122.41 (13)	H9A—C9—H9B	107.6
O4—C4—C5	124.89 (14)	C9—C10—C11	111.57 (13)
N3—C4—C5	112.70 (13)	C9—C10—H10A	109.3
C6—C5—F5	120.95 (13)	C11—C10—H10A	109.3
C6—C5—C4	122.57 (14)	C9—C10—H10B	109.3
F5—C5—C4	116.48 (13)	C11—C10—H10B	109.3
C5—C6—N1	121.32 (13)	H10A—C10—H10B	108.0
C5—C6—H6	119.3	C10—C11—H11A	109.5
N1—C6—H6	119.3	C10—C11—H11B	109.5
O7—C7—N1	116.83 (13)	H11A—C11—H11B	109.5
O7—C7—C8	123.69 (13)	C10—C11—H11C	109.5
N1—C7—C8	119.47 (12)	H11A—C11—H11C	109.5
C7—C8—C9	112.37 (12)	H11B—C11—H11C	109.5
C7—C8—H8A	109.1		
			
C6—N1—C2—O2	−179.04 (14)	F5—C5—C6—N1	−179.41 (12)
C7—N1—C2—O2	−3.7 (2)	C4—C5—C6—N1	0.2 (2)
C6—N1—C2—N3	0.63 (19)	C2—N1—C6—C5	0.1 (2)
C7—N1—C2—N3	175.95 (12)	C7—N1—C6—C5	−175.62 (13)
O2—C2—N3—C4	177.79 (14)	C6—N1—C7—O7	5.59 (19)
N1—C2—N3—C4	−1.9 (2)	C2—N1—C7—O7	−169.94 (13)
C2—N3—C4—O4	−178.30 (14)	C6—N1—C7—C8	−175.64 (12)
C2—N3—C4—C5	2.1 (2)	C2—N1—C7—C8	8.8 (2)
O4—C4—C5—C6	179.25 (14)	O7—C7—C8—C9	−6.9 (2)
N3—C4—C5—C6	−1.2 (2)	N1—C7—C8—C9	174.46 (12)
O4—C4—C5—F5	−1.1 (2)	C7—C8—C9—C10	−74.69 (17)
N3—C4—C5—F5	178.46 (12)	C8—C9—C10—C11	176.35 (13)

### Hydrogen-bond geometry (Å, °)

**Table:**

*D*—H···*A*	*D*—H	H···*A*	*D*···*A*	*D*—H···*A*
N3—H3···O4^i^	0.88	1.99	2.8588 (16)	170
C6—H6···O7	0.95	2.28	2.6102 (17)	100
C6—H6···O7^ii^	0.95	2.34	3.2266 (19)	154

Symmetry codes: (i) −*x*+2, −*y*+1, −*z*+2; (ii) −*x*, −*y*+1, −*z*+1.

## References

[bb1] Beall, H. D., Prankerd, R. J. & Sloan, K. B. (1997). *Drug Dev. Ind. Pharm.* **23**, 517–525.

[bb2] Jiang, A., Hu, S., Wang, Y. & Chen, Q. (1988). *Gaodeng Xuexiao Huaxue Xuebao*, **9**, 307–309.

[bb3] Lehmler, H.-J. & Parkin, S. (2000). *Acta Cryst.* C**56**, e518–e519.

[bb4] Nonius (1998). *COLLECT* Nonius BV, Delft, The Netherlands.

[bb5] Otwinowski, Z. & Minor, W. (1997). *Methods in Enzymology*, Vol. 276, *Macromolecular Crystallography*, Part A, edited by C. W. Carter Jr & R. M. Sweet, pp. 307–326. New York: Academic Press.

[bb6] Roberts, W. J. & Sloan, K. B. (1999). *J. Pharm. Sci.* **88**, 515–522. 10.1021/js980419b 10229642

[bb7] Sheldrick, G. M. (2008). *Acta Cryst.* A**64**, 112–122. 10.1107/S0108767307043930 18156677

